# Effects of Phloretin on Seedling Growth and Histochemical Distribution of Phenols, Polysaccharides and Lipids in *Capsella bursa-pastoris* (L.) Medik.

**DOI:** 10.3390/plants13141890

**Published:** 2024-07-09

**Authors:** Milica Đorđić, Dušica Janošević, Dijana Smailagić, Nevena Banjac, Slavica Ninković, Mariana Stanišić, Milena Trajković

**Affiliations:** 1 Department of Plant Physiology, Institute for Biological Research “Siniša Stanković”—National Institute of the Republic of Serbia, University of Belgrade, Bulevar Despota Stefana 142, 11108 Belgrade, Serbia; milica.djordjic@ibiss.bg.ac.rs (M.Đ.); dijana.smailagic@ibiss.bg.ac.rs (D.S.); mitic.nevena@ibiss.bg.ac.rs (N.B.); slavica@ibiss.bg.ac.rs (S.N.); mariana.stanisic@ibiss.bg.ac.rs (M.S.); 2 Faculty of Biology, University of Belgrade, Studentski trg 16, 11000 Belgrade, Serbia; dusjan@bio.bg.ac.rs

**Keywords:** allelopathy, apple, chlorophyll, dihydrochalcones, *Malus*, pectin, phytotoxic effect, starch

## Abstract

The present study evaluates the phytotoxic effects of phloretin, a prevalent secondary metabolite of apple trees, on the broadleaf weed *Capsella bursa-pastoris* (L.) Medik. known for its resistant myxospermous seeds that form a long-lasting soil bank. The results indicate a significant, dose-dependent inhibitory effect of phloretin on the growth and morphological parameters of weed seedlings grown in vitro. Although the applied phloretin concentrations (250–1000 µM) were not lethal to the *C. bursa-pastoris* seedlings after two weeks, the metabolism of the seedlings was impaired, resulting in an accumulation of lipid droplets in the root tips and root hairs. Histochemical analysis shows deposits of phenols in the root epidermal cells, which are probably aggregates of phloretin or its metabolic derivatives. The accumulation of pectin in the cell walls of root border cells in phloretin-treated seedlings indicates an attempt to reduce the uptake of phloretin and reduce its concentration in the cells. Inhibition of shoot growth associated with chlorosis and reduced photosynthetic pigment content is a consequence of seedling exposure to phloretin. This study provides a basis for further evaluation of phloretin as a new bioherbicidal compound and for elucidating the mechanism underlying its phytotoxic activity.

## 1. Introduction

Weeds adversely affect the growth and development of crops, which ultimately leads to reduced yields and high economic losses [1]. The extensive use of synthetic herbicides for weed control is a cause of public concern for human health and environmental protection [2]. In addition, the prolonged use of herbicides with the same mode of action (MOA) leads to herbicide resistance in weeds that no longer respond to these MOAs. Given the increasing concern about this problem, the development of weed control strategies based on natural compounds with novel mechanisms of action is essential [1].

Phloretin [3-(4-hydroxyphenyl)-1-(2,4,6-trihydroxyphenyl)-1-propanone] and its glucoside, phlorizin (phloretin-2’-O-glucoside), are classified as dihydrochalcones, which represent a unique subgroup of phenolic compounds with limited occurrence in the plant kingdom [3]. Dihydrochalcones are specific to species of the genus *Malus*, where they are the predominant secondary metabolites, accounting for up to 97% of total phenolics in leaves and comprising about 10–20% of DW leaf tissue [4,5].

Phloretin is a recognized phenolic substance from apple trees with beneficial properties for human health [6,7]. It has antidiabetic and anticancer activity [8,9], cytoprotective and anti-inflammatory activity [10,11] and various bioactive functions, including memory improvement [12], inhibition of lipid peroxidation [13], prevention of bone loss [14] as well as the antibacterial and antifungal activity [15]. Despite these recognized benefits, knowledge about the effect of phloretin on plant species is very scarce. Börner (1960) [16] was the first to point out the autotoxic properties of phloretin and phlorizin and their association with ARD (“Apple Replant Disease”), a condition characterized by slow growth, a reduced root system and low yields in apple trees planted on the sites of previous apple orchards. Phloretin and phlorizin, released into the soil by apple roots or generated through the decomposition of fallen leaves, bark, roots, fruit or other tree parts, can be considered as key factors contributing to the manifestation of ARD [17,18,19,20,21]. However, the mechanisms of the negative effects of phloretin on the newly planted apple trees are not yet fully understood.

The phytotoxicity of phloretin to other plant species was first reported by our research group in 2019 and 2022 [22,23], suggesting the potential use of phloretin as an eco-friendly bioherbicidal substance. Studies on the model plant Arabidopsis [*Arabidopsis thaliana* (L.) Heynh] [23] showed that phloretin is a potent growth inhibitor that causes severe morphological abnormalities and agravitropic responses of seedlings. This behavior is the consequence of a disruption of the auxin metabolome profile in roots, with particularly increased levels of indole-3-acetic acid (IAA) and oxoindole-3-acetic acid (OxIAA), accompanied by an altered expression of genes involved in auxin biosynthesis and polar auxin transport (PAT), as well as auxin accumulation in the lateral parts of root tips. A disturbed auxin homeostasis in the roots leads to a strongly reduced length of the meristematic and elongating root zones and to a reduced starch content in the root columella cells [23].

Shepherd’s purse [*Capsella bursa-pastoris* (L.) Medik.] is a small, annual, ruderal flowering plant belonging to the Brassicaceae family. It is a common weed on agricultural land, roadsides and meadows. It has a long-lasting seed bank in the soil and a short generation time, so that it can quickly colonize disturbed soils, such as agricultural fields, and produce several generations each year. It thrives in various climatic regions from northern countries to the Mediterranean and North Africa [24]. It also survives in arid desert environments due to its myxospermous seeds, which, after hydration, form a voluminous mucilaginous sheath composed mainly of polysaccharides of the plant cell wall (pectin, hemicellulose and cellulose) deposited during development in the cells that comprise the seed coat [25,26]. A selection advantage of myxospermous seeds probably lies in better germination [27] and/or better contact and adhesion to soil particles [28]. The viability of *C. bursa-pastoris* seeds under different environmental conditions and their rapid colonization characteristics make this species a good candidate for testing the phytotoxic potential of phloretin as a new prospective herbicidal compound.

The aim of the presented study is to evaluate the potential of phloretin to inhibit germination and growth of *C. bursa-pastoris*. The focus is on the effect on morphological parameters of the seedlings and the histochemical redistribution of primary and secondary metabolites such as phenols, starch, pectin and lipids in the roots as well as the content of photosynthetic pigments in the shoots after phloretin treatment. The results obtained in this study indicate a significant dose-dependent inhibition of seedling growth and changes in the distribution of phenols, pectin and lipids in the roots. Inhibition of shoot growth associated with chlorosis inevitably leads to an ineffective photosynthetic process in phloretin-treated seedlings.

## 2. Results

### 2.1. The Effect of Phloretin on the Weed Species Capsella bursa-pastoris L. In Vitro Growth

#### 2.1.1. Germination of *C. bursa-pastoris* Seeds Grown on a Medium with Different Concentrations of Phloretin

**Figure 1:**
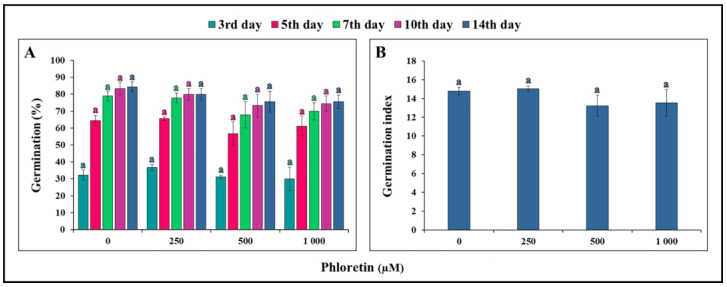
Germination of *C. bursa-pastoris* seeds on ½MS medium with different concentrations of phloretin (0, 250, 500 and 1000 μM) in in vitro culture. (**A**) Percentage of germination during 14-day period; (**B**) germination index at 14th day of seedling growth. Values represent means ± SE (standard error) of 30 seeds per each treatment repeated three times (n = 90). Values denoted by the same letter of the same color are not significantly different at *p* ≤ 0.05 per Fisher’s least significant difference (LSD) test.

During the 14-day period, seeds germinated asynchronously both on the control medium and on the media enriched with different concentrations of phloretin (0–1000 μM) (Figure 1A). The majority of seeds (approximately 65%) germinated within the first 5 days after light exposure, regardless of phloretin treatment. After this time, a small number of seeds had the potential to germinate. The germination index was also not affected by phloretin, and no statistically significant difference was found between the treatment and control groups (Figure 1B). Overall, it can be concluded that phloretin had no negative effect on the germination of *C. bursa-pastoris* seeds.

#### 2.1.2. Morphological Characteristics of *C. bursa-pastoris* Seedlings

**Figure 2:**
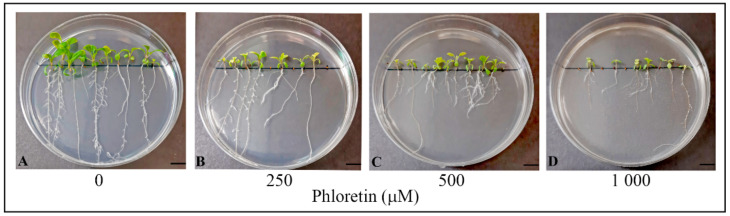
*C. bursa-pastoris* seedlings after 14 days of vertical growth in in vitro culture on ½MS nutrient medium with different concentrations of phloretin. Seedlings on the medium (**A**) without phloretin—control; (**B**) with 250 μM phloretin; (**C**) 500 μM phloretin; and (**D**) 1000 μM phloretin. Bar = 10 mm.

Characterization of the morphological parameters of *C. bursa-pastoris* seedlings grown on nutrient media enriched with different concentrations of phloretin (0–1000 μM) during a 14-day period indicated overall growth inhibition, which intensity depended on the concentration of phloretin applied (Figure 2). Visual changes in the color of leaves and roots were observed in all treatments after 14 days. Compared to the control (Figure 2A), the treatments with higher phloretin concentrations (500 and 1000 μM) showed more pronounced leaf chlorosis and root browning (Figure 2C,D).

**Figure 3:**
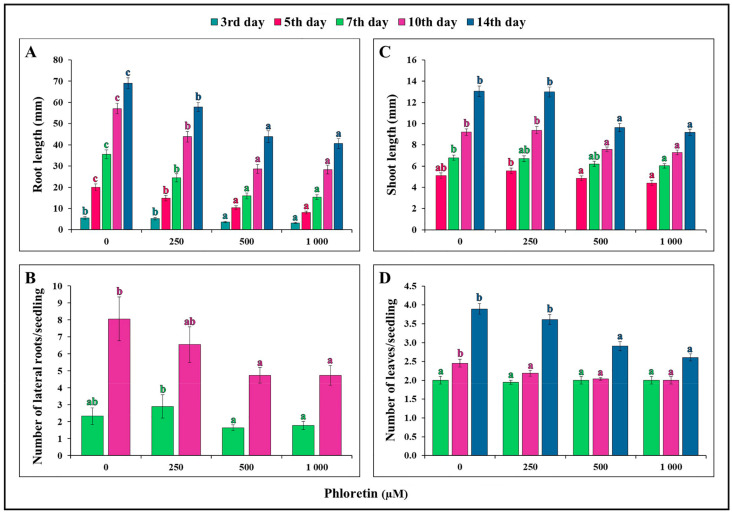
Phloretin effects on *C. bursa-pastoris* seedlings during 14 days of vertical growth in in vitro culture: (**A**) root length (mm); (**B**) number of lateral roots per seedling; (**C**) shoot length (mm); and (**D**) number of leaves per seedling. Values represent means ± SE of 30 seedlings per each treatment repeated three times (n = 90). Values denoted by the same letter of the same color are not significantly different at *p* ≤ 0.05 per Fisher’s least significant difference (LSD) test.

**Figure 4:**
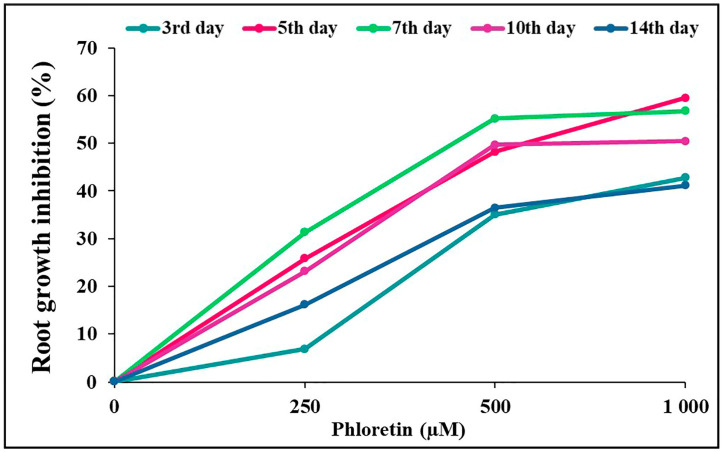
Dose-dependent curves of phloretin-induced root growth inhibition of *C. bursa-pastoris* seedlings during 14 days of vertical growth in in vitro culture.

During the 14-day growth period, we observed that phloretin had the strongest dose-dependent effect on root length development and growth (Figure 3A). After 3 days of seedling growth, the effect of phloretin on the roots was only evident on the media containing 500 and 1000 μM phloretin. A concentration of 250 μM became inhibitory after 5 days of seedling growth, but the effects were not pronounced compared to the higher concentrations. The highest inhibition of 59.5% occurred on the fifth day at 1000 μM (Figure 4).

Lateral roots began to grow from the axial root after 5 days of seedling growth on both the control medium and the phloretin-enriched medium (Figure 3B). The 250 µM phloretin concentration did not significantly affect the number of lateral roots even after 10 days of seedling growth compared to the control treatment without phloretin. The higher concentrations of phloretin (500 and 1000 μM) reduced the number of lateral roots per seedling after 10 days of growth.

The inhibitory effect of phloretin on shoot elongation of *C. bursa-pastoris* seedlings was less pronounced compared to roots (Figure 3C). Inhibition of shoot elongation was observed after 7 days of seedling growth at the highest concentration of phloretin (1000 μM). Subsequent observations after 10 and 14 days showed an inhibitory effect of phloretin on shoot elongation at concentrations of 500 and 1000 μM. Treatment with 250 μM phloretin did not result in a significant effect on shoot elongation of *C. bursa-pastoris*.

True leaves appeared after 5 days of seedling growth. There was no difference in the number of leaves between the control and the phloretin-treated seedlings after 7 days of growth (Figure 3D). The first significant difference in the number of leaves was observed at 10 days. After 14 days of seedling growth, only higher concentrations of phloretin (500 and 1000 μM) had a statistically significant inhibitory effect.

**Figure 5:**
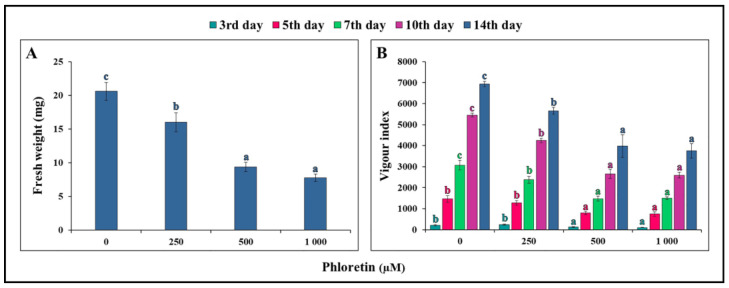
Phloretin effects on *C. bursa-pastoris* seedlings. (**A**) Fresh weight and (**B**) vigor index during 14 days of seedling growth in in vitro culture. Values represent means ± SE of 30 seedlings per each treatment repeated three times (n = 90). Values denoted by the same letter of the same color are not significantly different at *p* ≤ 0.05 per Fisher’s least significant difference (LSD) test.

Due to the phloretin-induced growth inhibition, the fresh weight of the seedlings was also reduced (Figure 5A). Seedlings grown on phloretin-free nutrient medium had an average mass of 20.6 mg, while seedlings grown on media containing 500 and 1000 μM phloretin had an average mass of 9.4 and 7.8 mg, respectively.

Phloretin also had a negative effect on the vigor index of the seedlings (Figure 5B). In the first days of seedling growth (third and fifth days), only higher concentrations of phloretin (500 and 1000 μM) resulted in significantly lower vigor indices compared to the control. The vigor indices were statistically significantly lower in all treatments after 7 days of seedling growth.

### 2.2. Photosynthetic Pigment Content in Shoots of C. bursa-pastoris

**Figure 6:**
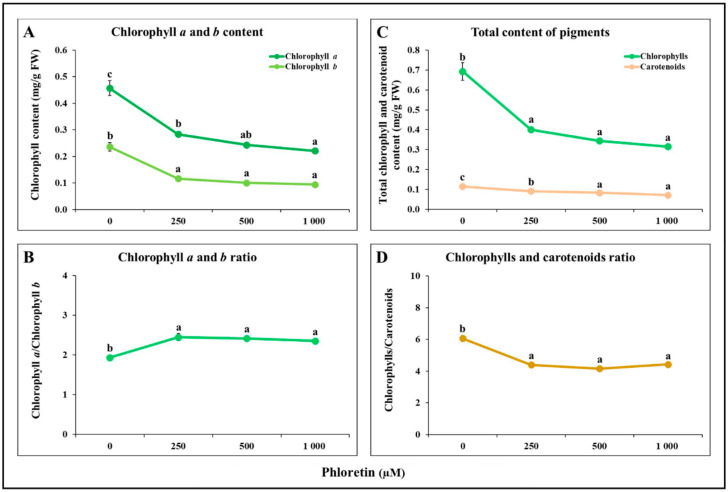
Phloretin effects on (**A**) chlorophyll *a* and *b* content; (**B**) chlorophyll *a* and *b* ratio; (**C**) total chlorophyll and carotenoid contents; and (**D**) chlorophylls and carotenoids ratio in *C. bursa-pastoris* seedling shoots after 14 days of seedling growth in in vitro culture. The results represent mean of three biological replicates. Different letters indicate statistically significant differences based on Fisher’s LSD test (*p* ≤ 0.05).

The content of chlorophylls and carotenoids was determined in the shoots after 14 days of seedling growth on a medium with different concentrations of phloretin (0, 250, 500 and 1000 µM). The results showed that the content of chlorophylls *a* and *b* in the shoots of *C. bursa-pastoris* decreased with increasing phloretin concentration (Figure 6A). Chlorophyll *b* was more affected by phloretin treatment and its content decreased more than that of chlorophyll *a*. After 14 days of treatment with the highest phloretin concentration (1000 µM), the content of chlorophyll *b* decreased by 60.16%, while the content of chlorophyll *a* decreased by 51.56% compared to the control. This resulted in an altered ratio of chlorophyll *a* to chlorophyll *b*, with chlorophyll *a* being less impacted in all phloretin treatments (Figure 6B). In addition, the concentration of total chlorophylls and total carotenoids decreased with increasing phloretin concentration. The inhibitory effect of phloretin was more pronounced for total chlorophylls than for carotenoids (Figure 6C). Accordingly, the ratio of total chlorophylls to total carotenoids decreased with increasing phloretin concentration in the nutrient medium (Figure 6D).

### 2.3. Histochemical Examination of C. bursa-pastoris Roots

Histochemical characterization of the roots of *C. bursa-pastoris* seedlings was performed after 14 days of in vitro culture on nutrient media with or without 500 μM phloretin. The study aimed to assess whether phloretin treatment induced changes in the histochemical composition of the roots, focusing on the presence of phenols, starch, pectin polysaccharides and lipids.

#### 2.3.1. Detection of Phenols

**Figure 7:**
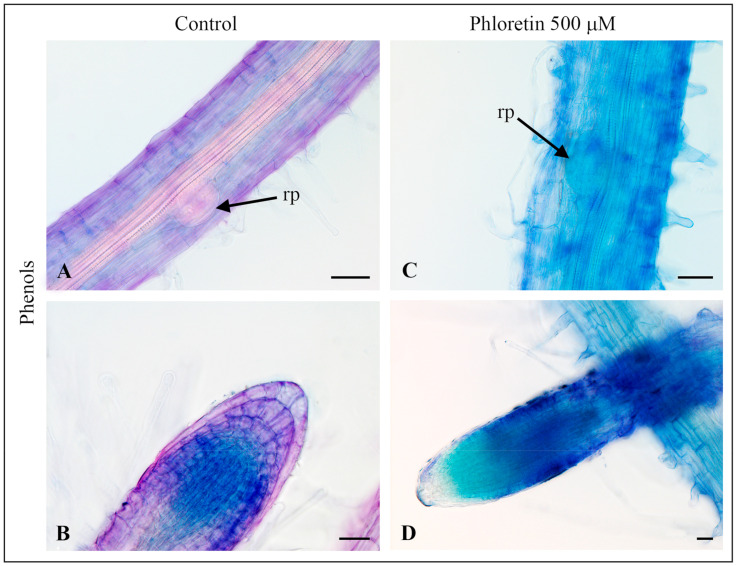
Toluidine Blue O staining of phenols in the roots of *C. bursa-pastoris* seedlings grown on the ½MS nutrient medium without (left) or with 500 µM phloretin (right) in culture in vitro for 14 days. Mature zone (**A**) and root apex (**B**) of control root; mature zone (**C**) and root apex (**D**) of phloretin-treated root. Bar = 50 µm (**A**,**C**) and 20 µm (**B**,**D**). rp—lateral root primordium.

Toluidine Blue O, a dye with the capability to stain various chemical compounds, ranging from phenols to urinoid polysaccharides and macromolecules containing phosphate groups, was employed for analysis. The staining patterns are distinctive: phenols show up in different shades of blue, polyphenols (lignin and tannins) in greenish-blue or light blue, pectin in light pink to purple and the primary wall (polyuronide polysaccharides) of epidermal and parenchymal cells in purple, while starch and cellulose remain unstained [29]. As shown in Figure 7A, the axial roots of control seedlings display bright purple coloration of polyuronide polysaccharides in the primary walls of epidermal cells in the mature zone and a light pink coloration of carboxylated polysaccharides/pectin in the vascular tissues and root primordia. The presence of phenols was not observed in the early stages of root primordia of control seedlings. Phenol synthesis begins during the development of young roots, but with maturation, synthesis decreases and remains mainly in the meristem zone of the root tip (Figure 7B).

Intensive blue coloration is noticeable in the axial roots and root hairs of seedlings cultivated with 500 µM phloretin (Figure 7C,D). This coloration is due to an intense accumulation of phenols, presumably phloretin and/or its metabolic derivatives, in the root cells. Only in the seedlings treated with phloretin, the root primordia show a light blue coloration in the early stages, indicating the presence of phenols (Figure 7C).

#### 2.3.2. Detection of Starch

**Figure 8:**
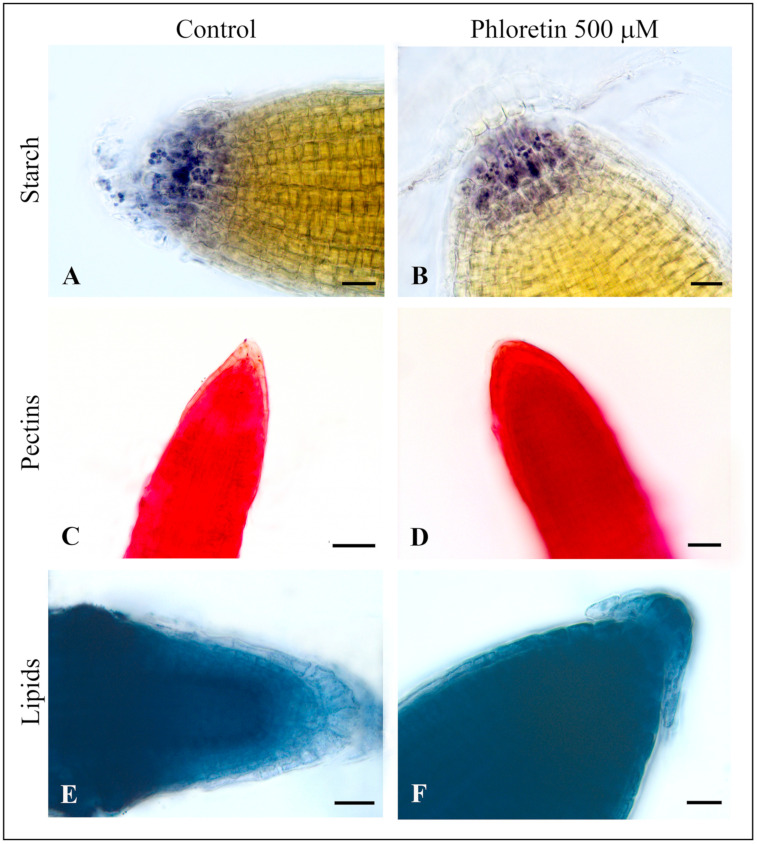
The root of *C. bursa-pastoris* seedlings grown on ½MS nutrient medium without (left) and with 500 µM phloretin (right) in culture in vitro for 14 days. Iodine–potassium iodide (IKI) staining of starch in the root tips of control (**A**) and phloretin-treated root (**B**). Bar = 20 µm (**A**,**B**). Rhutenium red staining of pectin in the root tips of control (**C**) and phloretin-treated root (**D**). Bar = 50 µm (**C**,**D**). Sudan Black B staining of lipids in the root tips of control (**E**) and phloretin-treated seedling (**F**). Bar = 20 µm (**E**,**F**).

The dye iodine–potassium iodide (IKI) colors starch blue to black, with newly synthesized starch being colored red–purple, short starch chains red–brown and long starch chains dark blue. Our results indicate the presence of starch in the apex of the axial root. Starch grains are present in the root cap of both control and phloretin-treated *C. bursa-pastoris* seedlings (Figure 8A,B), indicating that phloretin treatment did not significantly alter the content and distribution of starch. 

#### 2.3.3. Detection of Pectin

Ruthenium red, a dye that stains pectin pink to red, shows that the top layer of columella cells (root border cells) in control seedlings has a very low pectin content, as this area was very light red in color (Figure 8C). In contrast, the root border cells of phloretin-treated seedlings show an intense red coloration, indicating a high pectin content of these elements (Figure 8D). In addition, the overall color of the phloretin-treated roots appeared to be more intense than the color of the control roots. The deepest red color was abundant in the central parts of the treated roots, in the area belonging to the cortex. This indicated that the phloretin-treated roots contained more pectin compared to the control roots.

#### 2.3.4. Detection of Lipids

Staining with Sudan Black B dye clearly shows the differences in the presence of lipids in the roots of *C. bursa-pastoris* seedlings grown on nutrient medium without (control) and with 500 μM phloretin. The absence of dark blue coloration in the root apex of control seedlings indicates that the roots do not normally accumulate lipids in the columella cells (Figure 8E). The gradual change in color intensity from the columella and meristematic zone to the more mature parts of the root clearly indicates that lipids are only sparsely stored in the root apex and are mainly concentrated in the stele.

In contrast, the dark blue coloration of the root columella cells in the phloretin-treated seedlings indicates an intense accumulation of lipids (Figure 8F). The intensity of blue coloration was higher in the treated roots, and the distribution was almost the same in all parts of the roots, in contrast to the control roots. The cells of the phloretin-treated plants were barely visible and hardly recognizable due to the very high intensity of the dark blue coloration. Only the columella cells were lighter blue in color, but still more intense than in the control plants.

In addition, the granular structure of the root hairs and the epidermal root cells from which the root hairs develop indicate an increased secretion of lipid compounds in these areas of the phloretin-treated roots (Supplementary Material Figure S1).

## 3. Discussion

### 3.1. The Effect of Phloretin on Seed Germination and Seedling Growth in C. bursa-pastoris

Phloretin was recently recognized by our research group as a potent allelochemical from apple trees that has a significant phytotoxic effect on the model plant *A. thaliana* by disrupting the homeostasis of the plant hormone auxin, leading to growth stagnation and abnormal gravitropic responses [23]. With the idea to develop a new eco-friendly herbicide solution based on the phytotoxic properties of phloretin, in this study we investigate the effect of phloretin on shepherd’s purse [*Capsella bursa-pastoris* (L.) Medik.], a small broadleaf weed of the Brassicaceae family. *C. bursa-pastoris* has a long-lived seed bank in the soil and a short generation time. It is known to rapidly colonize agricultural fields, roadsides and meadows, producing several generations each year [30]. The high viability of the myxospermic seeds of *C. bursa-pastoris* under different environmental conditions and its rapid colonization ability make this species a suitable candidate for testing the phytotoxic potential of phloretin.

To evaluate the phytotoxic effect of phloretin on *C. bursa-pastoris*, the germination rate and morphological parameters of seedlings were studied under different concentrations of phloretin (from 0 to 1000 μM) during a 14-day period. It was found that phloretin treatment had no significant negative effects on the seed germination of this species (Figure 1A). This is in line with the results of our research group [23], where phloretin had no effect on seed germination of *A. thaliana* regardless of its concentration. Various studies have shown that allelochemicals actually have a stronger effect on the growth of treated plants than on the germination process itself. For example, the water extract of *Tithonia diversifolia* had no negative effect on the seed germination of maize, but significantly inhibited the root length of maize seedlings [31]. Consistent with this, the detrimental effect of phloretin on root growth of *C. bursa-pastoris* seedlings is evident as early as the third day of seedling growth after light exposure to nutrient media containing 500 and 1000 μM phloretin (Figure 3A). During the 14-day period, the effect of phloretin was strongly dose-dependent. But in contrast to *A. thaliana*, where the inhibition of root growth gradually increased with the duration of treatment and was highest at the end (75% and 86% of inhibition on the 10th day of treatment with 1000 µM and 1500 µM phloretin, respectively) [23], the percentage of inhibition in *C. bursa-pastoris* was highest at the beginning of treatment (59.5% on the fifth day with 1000 µM), but remained almost the same or even decreased in the following days (Figure 4). Negative effects of other chalcones on root growth of *A. thaliana* seedlings were demonstrated by Díaz-Tielas et al. (2012) [32], who reported that trans-chalcone at a concentration of 12.5 μM resulted in 30% inhibition compared to the control, while treatment with a concentration of 1200 μM caused a remarkable 94% inhibition of root growth.

Application of phloretin also resulted in a reduction in the number of lateral roots in *C. bursa-pastoris* seedlings, particularly evident at concentrations of 500 µM and 1000 μM (41.5% and 41.6%, respectively, after 10 days of treatment) (Figure 3B). Similarly, phloretin inhibited the development of lateral roots in *A. thaliana* seedlings with a higher percentage of inhibition at concentrations of 500 µM and 1000 µM after 10 days of treatment (75.6% and 58.9%, respectively) [23].

The differences in the phytotoxic potential of phloretin compared to these two related species were again demonstrated in the case of shoot growth inhibition. Inhibition of shoot growth increased with the duration of treatment, reaching the highest value of 29.7% in 14-day-old seedlings cultivated on media containing 1000 μM phloretin (Figure 3C). Treatments of the same duration and the same phloretin concentrations had almost two times stronger inhibitory effect on shoot growth in *A. thaliana* seedlings (48.1% at 1000 μM phloretin after 14 days) [23] compared to *C. bursa-pastoris*. Our results also showed a negative effect of phloretin on leaf formation, which was most evident after 14 days of treatment with higher concentrations of phloretin (500 and 1000 μM) (Figure 3D). The leaves not only showed a reduced number and size, but were also more yellowish compared to the control. This is in line with similar observations of the effects of phloretin on *A. thaliana* seedlings as reported by Stanišić (2019) [33] and Smailagić et al. (2022) [23].

Overall, the results obtained confirmed that phloretin has a stronger inhibitory effect on root growth and development of the tested plant species than on the growth of shoots. This observation is consistent with the known sensitivity of the root system to allelochemicals due to direct exposure to toxic compounds and the high permeability of root tissues [34,35,36,37]. These results are in accordance with previous studies emphasizing the inhibitory effect of phytotoxic compounds on root growth. The allelopathic effect of root exudates of *Malus hupehensis* Rehd. was found to exert a stronger inhibitory influence on root growth of the same species than on shoot growth. Other researchers also observed a stronger effect of allelochemicals on root growth and below-ground biomass [38,39,40].

### 3.2. Effects of Phloretin on the Content of Photosynthetic Pigments in Seedling Shoots

Plants are photosynthetic organisms that use carbon dioxide (CO_2_) and water (H_2_O) with the help of light energy to produce organic molecules, releasing oxygen (O_2_) in the process. Photosynthetic pigments, especially chlorophylls, capture the light energy, while carotenoids, as secondary pigments, absorb the excess energy from the sun. The amount of photosynthetic pigments in plants is a critical parameter indicating the efficiency of photosynthesis. The effect of allelochemicals on photosynthesis generally consists of inhibition or damage to the pigment synthesis machinery and accelerated degradation of photosynthetic pigments [41]. The water extract of the allelopathic plant *Tithonia diversifolia* was found to reduce the content of chlorophylls *a* and *b* in maize seedlings [31]. Dayan et al. (1999) [42] emphasized that artemisinin, a sesquiterpenoid lactone found in *Artemisia annua*, also reduced chlorophyll content in the treated plants. Similar results were observed with the water extract of *Artemisia argyi* in rice seedlings [43].

Our results illustrate that the contents of chlorophylls *a* and *b* in the shoots of *C. bursa-pastoris* seedlings decreased with increasing phloretin concentration (Figure 6A). Chlorophyll *b* is more sensitive to phloretin treatment, resulting in an increase in the chlorophyll *a*/*b* ratio in all phloretin treatments (Figure 6B). Since there is evidence that chlorophyll *b* can be converted to chlorophyll *a* during chlorophyll degradation [44,45], the same process may be occurring in phloretin-treated seedlings, resulting in a more rapid decrease in chlorophyll *b* content than chlorophyll *a* content and thus a higher chlorophyll *a*/*b* ratio.

In addition, it can be observed that the total carotenoid content also decreases with increasing phloretin concentration, but is less affected than the total chlorophyll content (Figure 6C). Overall, the reduced values of the ratio of total chlorophyll to total carotenoids in *C. bursa-pastoris* seedlings under phloretin treatment (Figure 6D) indicate stress, senescence and damage to the photosynthetic apparatus [46]. A similar effect of chalcones was also reported by Díaz-Tielas et al. (2014) [47], who demonstrated chlorosis in *Amaranthus retroflexus* and *Triticum aestivum* after treatment with trans-chalcones. The results of studies with a number of allelopathic compounds with different chemical basis indicate the same kind of effects [48,49,50,51], confirming that direct reduction in light absorption efficiency and inhibition of photosynthetic capacity are the common phytotoxic mechanisms for allelopathic interactions in plants.

### 3.3. Histochemical Analysis of Seedling Roots

The roots of 14-day-old *C. bursa-pastoris* seedlings grown on nutrient media without (control) or with 500 μM phloretin were subjected to histochemical analysis to determine whether phloretin treatment induced alteration in the chemical composition of the roots, focusing on the presence of phenols, starch, pectin polysaccharides and lipids.

The blue coloration visible in root cells after Toluidine Blue O staining of phenolics in phloretin-treated seedlings probably represents an accumulation of phloretin and/or its metabolic derivatives (Figure 7C,D). As a phenolic compound belonging to the dihydrochalcone group [3], phloretin actively interacts with various biological molecules, resulting in plants’ tendency to rapidly convert phloretin into more stable, soluble, glycosylated forms that can be actively transported into vacuoles [52,53].

It is known that the starch-filled amyloplasts in the columella cells of roots play an essential role in the first steps of root gravitropic stimulus perception [54]. Zhang et al. (2019) [55] demonstrated that the local auxin maximum/gradient within the root apex, which is generated by PIN-FORMED (PIN) directional auxin transporters regulates the expression of three key starch granule synthesis genes (*SS4*, *PGM* and *ADG1*), which in turn influence the accumulation of starch granules that serve as a statolith for gravity perception. It has been shown that many natural compounds of plant origin are able to affect root growth and gravitropic response in recipient plants by inhibiting starch synthesis and accumulation or by interfering with the positioning of starch grains in root columella cells. For example, artemisinin, a secondary metabolite produced by *Artemisia annua*, reduces the sensitivity of *A. thaliana* roots to gravitropic stimuli and leads to a reduction in the number of starch grains [56]. Similarly, narciclasine, an alkaloid isolated from the bulbs of *Narcissus tazetta*, induces defects in root gravitropism that correlate with a reduction in auxin transport and the accumulation of starch granules in the columella cells of *A. thaliana* roots [57]. Allelochemicals with chalcone structure were also found to affect starch reserves in the amyloplasts, thereby interfering with the normal gravitropic response in the recipient plants. For example, one week after the initiation of trans-chalcone treatment in *A. thaliana* seedlings, disorganized starch was observed in the amyloplasts, which completely resolved after two weeks of treatment [32]. Consistent with this, our research group has recently reported that phloretin significantly reduced the starch content in the root tips of *A. thaliana* seedlings compared to the control, contributing to the loss of normal gravitropic response and the occurrence of morphological deformations [23]. Although reduced and disorganized starch in the amyloplasts in the root tips of *C. bursa-pastoris* seedlings was expected, the results show that starch is present in the columella cells of the roots in both the control and the treatment (Figure 8A,B), suggesting that phloretin did not alter the spatial distribution of starch or the gravitropic response of the roots. The presence of starch is visible in the root apex of phloretin-treated roots even at the early stages of root development, i.e., in the root primordium. This indicates that the gravitropic response of this weed species is not as sensitive to phloretin as in *A. thaliana*, possibly due to the fact that it has developed various tolerance mechanisms during evolution that ensure its survival and competitive advantage over other species.

Ruthenium red is a cationic dye with six positive charges, which is able to form electrostatic bonds with the acidic groups of the polyuronic acids of pectin [58]. We used Ruthenium red dye to mark differences in the accumulation of pectin polysaccharides in the cell walls between control and phloretin-treated roots. The results of our study indicate excessive accumulation of pectin in the cell walls of the upper layer of columella cells (root border cells) of roots of phloretin-treated *C. bursa-pastoris* seedlings (Figure 8D). Nagayama et al. (2019) [59] reported a significant increase in pectin in the root border cells of rice (*Oryza sativa*) roots under aluminum (Al) stress. The parallel experiments on the WT and sensitive to Al rhizotoxicity (*star1*) mutant rice plants showed that the accumulation and distribution of pectin in the root tips of WT plants after Al exposure contributed to the development of Al tolerance, presumably due to the Al-binding properties of the negatively charged carboxyl groups in the pectin molecule, as previously described [60,61]. In this sense, the accumulation of pectin in the root border cells of *C. bursa-pastoris* could be a way to prevent the uptake of phloretin and reduce its concentration in the cells through direct interaction with pectin. It is known that the formation of covalent bonds between polysaccharides, such as pectin and polyphenols, is possible [62,63,64]. In addition, polysaccharides are able to form multiple hydrogen bonds and hydrophobic interactions with polyphenols, resulting in extremely stable polysaccharide–polyphenol complexes [65,66]. Fernandes et al. (2019) [67] reported direct interactions between phlorizin and pectic polysaccharides—arabinans from sugar beet. The authors suggested that hydrophobic domains formed by the entanglement of the polymers entrap the phlorizin molecule in a mechanism similar to that of dextrins [68]. To support our assumption that plants strengthen their cell walls with pectin when exposed to stress, we found that some other allelochemicals also induced excessive accumulation of pectin in the roots of target plants. Citral, a volatile monoterpene compound from the essential oil of various aromatic plants, significantly altered the area and intensity of Ruthenium red coloration of *A. thaliana* roots compared to the control [69]. The duration of citral treatment increased pectin deposition in the walls of all root cells.

Abiotic stress has been found to lead to an accumulation of lipids in the seeds and vegetative tissues of various plant species [70] and in the cells of microalgae [71]. However, the changes in lipid accumulation and composition are strongly influenced by the nature of the stressor and the plant species. An accumulation of triglycerides in the roots was observed under salt stress in *Chloris gayana* Kunth [72] and in the roots of cucumber seedlings treated with DIBOA (2,4-dihydroxy-1,4(2H)-benzoxazin-3-one), the allelochemical from rye (*Secale cereale*) [73]. Olsson et al. (1996) [74] reported increased levels of triacylglycerol and free fatty acids as well as an altered ratio of monogalactosyl-diacylglycerols/digalactosyl-diacylglycerols in *Lotus corniculatus* under drought stress. The excessive accumulation of lipids in the tips of axial roots and root hairs of phloretin-treated *C. bursa-pastoris* seedlings (Figure 8F) indicates inhibited lipid degradation in the cells and/or insufficient utilization of energy for cell growth and proliferation due to growth stagnation. Although phloretin at the concentrations tested is not sufficient to cause *C. bursa-pastoris* seedling death over the two weeks, seedling metabolism is adversely affected, resulting in a feedback loop with reduced degradation/consumption of lipids at one end and slowed seedling growth at the other. A similar impaired lipid degradation and growth arrest in response to the allelochemicals gramin and hordenin from barley (*Hordeum vulgare* L.) was observed in the root cells of white mustard (*Sinapis alba*) [75].

## 4. Materials and Methods

### 4.1. Plant Materials and Experimental Design

The seeds of shepherd’s purse [*Capsella bursa-pastoris* (L.) Medik.] were collected in 2021 from meadows of Belgrade suburb (44°45′03.3″ N, 20°25′05.6″ E). Seeds were sterilized in a solution mixture (1:1) of 70% (*v*/*v*) ethanol (Zorka Pharma-Hemija DOO, Šabac, Serbia) and 30% (*v*/*v*) commercial bleach containing at least 3.5% active chlorine (Lavazza DOO, Novi Sad, Serbia) for 45 s. The seeds were then rinsed four times for 1 min in sterile deionized water. The seeds were placed in Petri dishes containing solid ½MS nutrient medium (half-concentrated mineral salts [76] and vitamins [77], enriched with 30 g/L sucrose, 8 g/L agar (Institute of Virology, Vaccines, and Serums “Torlak”, Belgrade, Serbia) and 0.1 g/L myo-inositol (Sigma-Aldrich, St. Louis, MO, USA)). The pH of the medium was adjusted to 5.8 using a pH meter (Edge pH manual, Hanna Instruments, Belgrade, Serbia) before sterilization. Phloretin (Sigma, St. Louis, MO, USA) was added to the sterilized and cooled medium in the laminar chamber at concentrations of 0, 250, 500 and 1000 μM. The stock solution of phloretin was prepared in dimethyl sulfoxide (DMSO; Duchefa Bio-chemie, Haarlem, The Netherlands). DMSO was added in the control medium to the same final concentration as in the phloretin-enriched medium [0.1% (*v*/*v*)]. The Petri dishes containing the sterilized seeds were partially sealed with tape to prevent excessive ethylene accumulation. After a 3-day cold stratification at 4 °C in the dark, the Petri dishes were transferred to the growth chamber with long-day conditions (16 h light/8 h darkness) and kept vertically in racks at 22 ± 2 °C. Fluorescent lamps (Tesla, Pančevo, Serbia; 65 W, 4500 K, with a light flux density of 35 ± 2 μmol/m^2^s) were used as the light source. For each treatment (phloretin concentration), three Petri dishes containing 10 seeds each were prepared. The experiment was repeated three times (n = 90) using a randomized design.

### 4.2. Germination Rate and Morphological Observations

To quantify seed germination and morphological parameters of the *C. bursa-pastoris* seedlings, the Petri dishes were examined under a stereomicroscope (Carl Zeiss, Göttingen, Germany) on the 3rd, 5th, 7th, 10th and 14th days. Root and shoot lengths as well as the number of lateral roots were determined using ImageJ software (ImageJ 1.53t, National Institutes of Health, Bethesda, MD, USA). Shoot length was measured from the hypocotyl-root junction to the top of the longest cotyledon/leaf, while the number of leaves (without cotyledons) were counted under a stereomicroscope (Carl Zeiss). The fresh weight of the seedlings was determined on day 14 using an analytical balance (ABJ 220-4M, Kern & Sohn, Balingen, Germany).

The percentage of root growth inhibition was calculated using the following formula [23]:

Inhibition percentage (%) = [1 − (value on the phloretin enriched medium/mean value on the control medium)] × 100

Additionally, seed germination percentage, germination index and vigor index were calculated using the following formulas [78]:

Germination percentage (%) = number of germinated seeds/total number of seeds × 100

Germination index = number of germinated seeds/days of the first count + … + number of germinated seeds/days of the final count

Vigor index = ∑ (root length + shoot length) × % germination

### 4.3. Photosynthetic Pigment Content Assessment

Shoots (50 mg FW) of *C. bursa-pastoris* seedlings grown for 14 days on the control medium and the phloretin-enriched medium (250, 500 and 1000 µM) were cut from the roots and immediately frozen in liquid nitrogen and stored at −80 °C until further analysis. Samples were collected in three biological replicates for each treatment. The plant material was grounded in liquid nitrogen. After addition of 1 mL of 80% acetone, the samples were centrifuged at 12,000× *g* for 10 min in an Eppendorf 5430R centrifuge (Hamburg, Germany). The supernatants were stored in the absence of light until spectrophotometric measurements (Agilent 8453 UV–visible spectrophotometer, Santa Clara, CA, USA). The samples were diluted with 80% acetone at a ratio of 3:1 (*v*/*v*) to obtain absorbance values in the acceptable range of 0.3–0.8 at 470, 647 and 663 nm. The measurements were performed in three technical replicates. Pigment concentrations were calculated using the following formulas [79] and expressed in mg/g of sample FW:

Chlorophyll *a*: C*a* (μg/mL) = 12.25 × A_663_ − 2.79 × A_647_

Chlorophyll *b*: C*b* (μg/mL) = 21.50 × A_647_ − 5.10 × A_663_

Carotenes and xanthophylls: C (*x + c*) (μg/mL) = (1000 × A_470_ − 1.82 × C*a* − 85.02 × C*b*)/198

Chlorophyll (*a* + *b*) (μg/mL) = 7.15 × A_663_ + 18.71 × A_647_

Ratio of chlorophyll *a* and *b* = C*a*/C*b*

Ratio of total chlorophylls and carotenoids = Chl (*a* + *b*)/C (*x + c*)

C = (C_x_ × V × R)/m

where C_x_ is concentration in μg/mL, V is extract volume in mL (1 mL), R is dilution factor (3) and m is sample weight in mg (50 mg).

### 4.4. Statistical Analyses

Data from measurements of morphological parameters and pigment content analysis obtained on the 3rd, 5th, 7th, 10th and 14th days of seedling growth were analyzed separately using one-way ANOVA. Fisher’s LSD (least significant difference) post hoc test was used to determine statistically significant differences between means, with a significance level *p* ≤ 0.05. Data were analyzed using Statistica12 statistical software (StatSoft, Inc., Tulsa, OK, USA).

### 4.5. Histochemical Analysis of C. bursa-pastoris

The seeds of *C. bursa-pastoris* L. were cultivated on the control medium or the ½MS nutrient medium supplemented with 500 μM phloretin, as described in Section 4.1. On day 14, seedling roots of the seedlings were carefully cut with a razor blade, and various root sections were subjected to histochemical analysis for the detection of phenols, starch, pectin and lipids. The samples were analyzed with a Carl Zeiss Axiovert microscope (Carl Zeiss GmbH, Göttingen, Germany).

#### 4.5.1. Staining of Phenols

Toluidine Blue O dye, as described by O’Brien et al. (1964) [80], was used for the detection of phenols and urinoid polysaccharides. An aqueous solution of Toluidine Blue O [0.05% (*w*/*v*)] was prepared in a 0.1 M phosphate buffer at pH 6.8. Root sections were rinsed with distilled water to remove agar residues and then immersed in the dye for 2 min, followed by rinsing with distilled water. The root samples were then fixed in a drop of glycerol.

#### 4.5.2. Detection of Starch

For the detection of starch, a solution of iodine (I_2_) and potassium iodide (KI) in water (IKI solution) [58] was used. The dye was prepared by dissolving 2 g of potassium iodide (KI) in 100 mL of water and adding 0.2 g of iodine to the prepared solution. Since the dye was unstable, the solution was stored in a dark bottle. The root sections were incubated in the IKI solution for 5 min. After staining, the plant material was briefly rinsed in distilled water and fixed in a drop of glycerol for microscopic observation.

#### 4.5.3. Detection of Pectin

Ruthenium red binds selectively to acidic polysaccharides such as pectin, resulting in an intense red coloration. This specific staining reaction enables the identification of pectin in the samples. A 0.02% aqueous solution of Ruthenium red was used to detect pectin in *C. bursa-pastoris* root sections [58]. Root samples were immersed in the dye for 5–10 min, then rinsed in distilled water and transferred to a glass slide in a drop of glycerol.

#### 4.5.4. Staining of Lipids

The presence of lipids (phospholipids, sterols and neutral triglycerides) in the root tips was detected using the Sudan Black B staining method [81]. In brief, 0.7 g of Sudan Black B dye was dissolved in 100 mL of 70% ethanol. The dye was filtered before use and stored in a dark bottle. After rinsing with distilled water to remove the residual agar medium, the root sections were immersed in 50% ethanol for 3 min and then in Sudan Black B dye for 20 min, in the dark at room temperature. Finally, the stained root sections were rinsed in 50% ethanol for 1 min. All root samples were placed on a glass slide and fixed in a drop of glycerol. For technical control of the staining method, the root sections were immersed in a solution of methanol, chloroform, water and hydrochloric acid (66:33:4:1) for 3 h in the dark at room temperature and then rinsed in 50% ethanol for 3 min. The root sections were then subjected to the staining procedure described above.

## 5. Conclusions

The human health-promoting properties of phloretin on the one hand and its phytotoxic effect on the other make it an ideal candidate for the development of an eco-friendly bioherbicidal solution. The results obtained show that phloretin does not inhibit seed germination of *C. bursa-pastoris*, but significantly reduces seedling growth. Although phloretin at the concentrations tested is not sufficient to cause the death of *C. bursa-pastoris* seedlings within two weeks, seedling metabolism is affected, leading to an accumulation of lipid droplets in the root tips and root hairs, presumably due to reduced lipid consumption. Histochemical analysis shows deposits of phenols in the roots, which are probably aggregates of phloretin or its metabolic derivatives. Excessive accumulation of pectin in the cell walls of root border cells of phloretin-treated seedlings indicates an attempt to reduce the uptake of phloretin and reduce its concentration in the cells. Inhibition of shoot growth, associated with chlorosis and reduced levels of photosynthetic pigments, inevitably leads to reduced efficiency of the photosynthetic process contributing to overall phloretin stress. This study provides a basis for further evaluation of phloretin as a new bioherbicidal compound and for elucidating the mechanism underlying its phytotoxic activity.

## Data Availability

All the data are in the manuscript.

## Supplementary Materials

The following supporting information can be downloaded at: https://www.mdpi.com/article/10.3390/plants13141890/s1, Supplementary Material Figure S1. The root of *C. bursa-pastoris* seedlings grown on ½MS nutrient medium with 500 µM phloretin in culture in vitro for 14 days. Sudan Black B staining of lipids in the epidermal root cells (A), and the root hairs of phloretin-treated seedling (B,C). Bar = 50 µm (A) and 20 µm (B,C).
