# Supplementary material for: Effects of Phloretin on Seedling Growth and Histochemical Distribution of Phenols, Polysaccharides and Lipids in Capsella bursa-pastoris (L.) Medik
Source: Plants (Basel). 2024 Jul 9;13(14):1890. doi: 10.3390/plants13141890 (PMC11280091; doi:10.3390/plants13141890)
Supplement: Supplementary file 1 [file plants-13-01890-s001.zip › plants-2961225-supplementary.pdf]

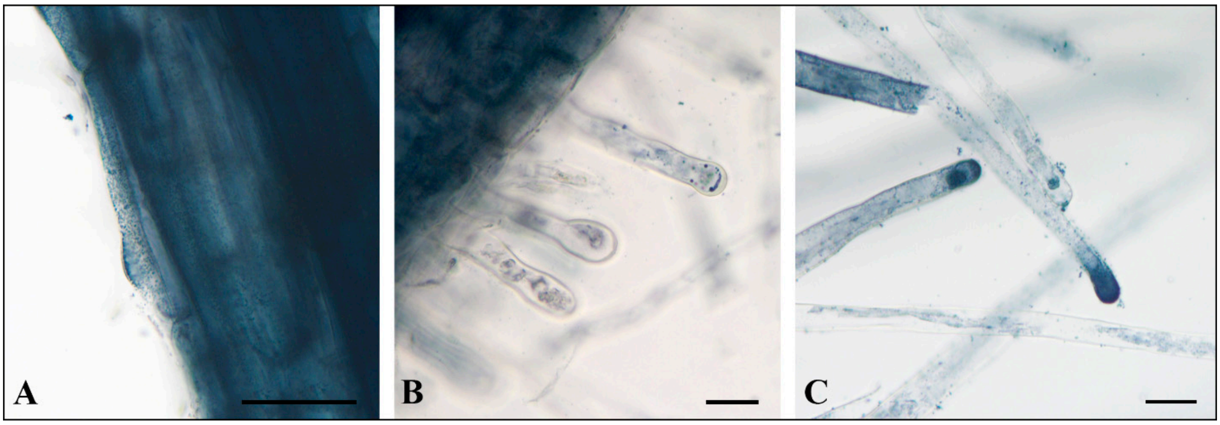

Supplementary Material Figure S1. The root of *C. bursa-pastoris* seedlings grown on ½MS nutrient medium with 500 µM phloretin in culture in vitro for 14 days. Sudan Black B staining of lipids in the epidermal root cells (**A**), and the root hairs of phloretin-treated seedling (**B**,**C**). Bar = 50 µm (**A**) and 20 µm (**B**,**C**).
